# SNR-Weighted Layer-Conditioned Magnetometer Array with Ellipsoid Calibration and Neural Network Initialization for Capsule Endoscopy Localization Under Asymmetric Sensor Visibility

**DOI:** 10.3390/s26175536

**Published:** 2026-08-31

**Authors:** Omid Yaghoobian, Khan A. Wahid

**Affiliations:** Department of Electrical and Computer Engineering, University of Saskatchewan, 57 Campus Drive, Saskatoon, SK S7N 5A2, Canada; khan.wahid@usask.ca

**Keywords:** magnetometer array, dipole localization, SNR-weighted fusion, ellipsoid calibration, asymmetric visibility, manifold-constrained estimation

## Abstract

Passive magnetic localization for wireless capsule endoscopy degrades under involuntary gastrointestinal motion: displacement toward one sensor layer simultaneously saturates near-side magnetometers while driving far-side sensors below the noise floor, a failure mode we term asymmetric layer visibility. Existing single-layer and multi-layer systems do not model this explicitly, and full-array Jacobian conditioning degrades progressively with displacement even where a layer-conditioned decomposition remains comparatively stable. We present a two-layer magnetometer array with three algorithmic contributions: per-sensor SNR gating excluding saturated or low-SNR sensors before optimization; layer-conditioned Levenberg–Marquardt estimation on a five-dimensional manifold via spherical parameterization; and SNR-weighted fusion with geodesic interpolation on $S^{2}$, combined with layer-wise ellipsoid calibration and neural network initialization. Eight LIS3MDL tri-axial magnetometers in a 15×15×30 cm^3^ two-layer array were validated across 150 Monte Carlo trials using a 4-DOF robotic arm. Mean positional error was 2.32±0.35 mm and orientational error 2.15±0.88° at 12.4 Hz, with 93.85% convergence success under displacement-induced asymmetric visibility events of 1.00–4.00 cm—over 70% reduction over single-layer and layer-agnostic baselines. Hardware generalization was confirmed across three magnetometer types. The SNR weight ratio also yields a passive discriminant between capsule displacement and global array motion, cross-validated AUC 0.924±0.045, requiring no additional hardware. Reported accuracy is specific to the LIS3MDL magnetometer used in this study.

## 1. Introduction

Wireless capsule endoscopy (WCE) is the primary modality for small-bowel visualization, a region inaccessible to push enteroscopy [1]. Despite its diagnostic value in obscure GI bleeding, Crohn’s disease, and polyposis syndromes, WCE lacks reliable real-time spatial localization. Current practice estimates position from capsule transit time multiplied by average speed—an approach with documented uncertainty exceeding 10 cm in the mid-jejunum [2].

This gap has three direct consequences for sensing system requirements. First, lesion co-registration for balloon enteroscopy requires sub-centimeter positional accuracy to determine the correct approach route [3]. Second, emerging robotic WCE platforms for active steering require real-time positional feedback at latencies below 200 ms [4]. Third, segmental transit analysis requires sufficient localization accuracy to distinguish jejunal from ileal transit [5]. These transit-time and timing-based approaches share a common limitation: none exploits a physical field that varies predictably and continuously with capsule position, which is precisely the property that makes magnetic localization a mechanistically distinct alternative to the indirect, elapsed-time-based methods above [1,2,3,4].

Magnetic localization is a candidate solution to this gap: unlike the transit-time estimation methods above, which infer position indirectly from elapsed time and average propulsion speed, embedding a permanent magnet in the capsule enables direct, continuous position recovery from external field measurements, motivating its investigation as an alternative to time-index-based approaches.

Passive magnetic localization—embedding a permanent magnet in the capsule and recovering position from external magnetometer measurements—is attractive because it requires no capsule-side power, no line-of-sight, and no ionizing radiation [2,6]. However, involuntary GI contractions abruptly shift capsule–sensor distances. The dipole field follows a $1/R^{3}$ law: a 2 cm displacement toward one sensor layer simultaneously saturates near-side sensors while driving far-side sensors below the noise floor. We term this *asymmetric layer visibility*.

Prior single-layer methods lose observability entirely when saturation occurs [7,8]. Multi-layer methods that treat all sensors as a unified array allow saturated measurements to corrupt the global optimization [6]. Neither approach treats asymmetric visibility as an explicit failure mode requiring dedicated system design. This gap motivates the present work.

The contributions of this paper are: (i) per-sensor SNR gating that excludes saturated and low-SNR sensors before LM begins, preventing ill-conditioned Jacobian rows from corrupting the solve; (ii) manifold-constrained layer-conditioned LM via explicit spherical parameterization (ϑ, φ), reducing the parameter space from 6D to 5D and eliminating post hoc renormalization bias; (iii) SNR-weighted visibility fusion with geodesic SLERP orientation interpolation on $S^{2}$, combined with layer-wise ellipsoid calibration and neural network initialization replacing exhaustive grid search; and (iv) comprehensive experimental validation across 150 Monte Carlo trials with hardware generalization across three sensor–magnet configurations, and a passive SNR weight asymmetry index δ discriminating capsule displacement from global array motion without additional hardware.

## 2. Literature Review

### 2.1. Magnetic Localization Systems: Technical Gaps

Passive magnetic localization, which recovers capsule position from the field of an embedded permanent magnet measured by external sensors, is the most compatible approach because it requires no capsule-side power and no ionizing radiation [2,6]. Prior systems can be grouped into three categories, each with a distinct technical limitation.

*Single-layer systems* have been the dominant approach. Song et al. [7] achieved 1.4 mm accuracy with an optimized eight-sensor planar array under static conditions only, and their reported sensitivity analysis showed accuracy degrading sharply once the capsule moved outside the central calibration volume, a limitation the present work addresses directly through layer-wise rather than global modeling. Zeising et al. [8] addressed wearable body motion via differential sensing but retained a single-layer architecture that loses observability under saturation; their differential approach compensates for rigid-body translation of the entire array relative to the body but provides no mechanism for sensor-level saturation within a single layer. Vedaei and Wahid [9] combined PSO-based initialization with LM in a nine-sensor array with relative motion evaluation, but without multi-layer architecture or asymmetric visibility treatment; their relative-motion protocol is the closest prior dynamic validation to the one used here, though it does not isolate displacement-induced saturation as a distinct failure mode. Shao and Guo [10] and Song et al. [11] proposed SNR-based array optimization and differential wearable tracking, respectively, both without multi-layer coverage; notably, Shao and Guo’s SNR-based sensor placement strategy operates at the array-design stage rather than at run time, which is the gap the per-sensor gating mechanism in this paper closes.

*Multi-layer systems* improve geometric coverage but have not addressed the failure mode that motivates this work. Shao et al. [6] developed a wearable 2-layer 16-sensor system but fused all sensors into a single optimization, allowing saturated measurements from one layer to corrupt the full-array estimate; their reported 10.0 mm accuracy reflects this vulnerability under the displacement conditions evaluated. Liu et al. [12] proposed a 96-sensor reconfigurable array at hardware complexity incompatible with wearable deployment, trading sensor-count redundancy for the observability robustness that architectural layer-conditioning achieves here with a fraction of the channel count. Hu et al. [13] and Son et al. [14] analyzed sensor count and 2D array configurations without evaluating relative motion or layer-specific failure modes, leaving open whether their reported static accuracy would hold under peristaltic-scale displacement.

*Temporal filtering approaches* including Extended Kalman Filters [15] and hybrid IMU/camera methods [16,17] improve trajectory continuity or robustness but do not address spatial observability loss from asymmetric saturation, and hybrid methods require capsule-side hardware modifications conflicting with the passive design constraint. These methods are complementary rather than competing: a temporal filter applied downstream of the present framework could further smooth the frame-level estimates produced here, particularly across the brief non-convergence windows discussed in Section 4.

### 2.2. Identified Gaps

Two gaps emerge from the above review. First, no prior method explicitly models asymmetric layer visibility—the simultaneous saturation of near-side sensors and low-SNR degradation of far-side sensors during involuntary GI displacement—as a system-level failure mode requiring dedicated architectural response. Second, evaluation under peristaltic-scale dynamic conditions is rare; most systems are validated only under static or slowly varying capsule positions [18]. This work directly addresses both gaps, and Section 5 returns to this comparison with quantitative results once the proposed framework has been described and validated.

## 3. Materials and Methods

### 3.1. System Model

**Figure 1:**
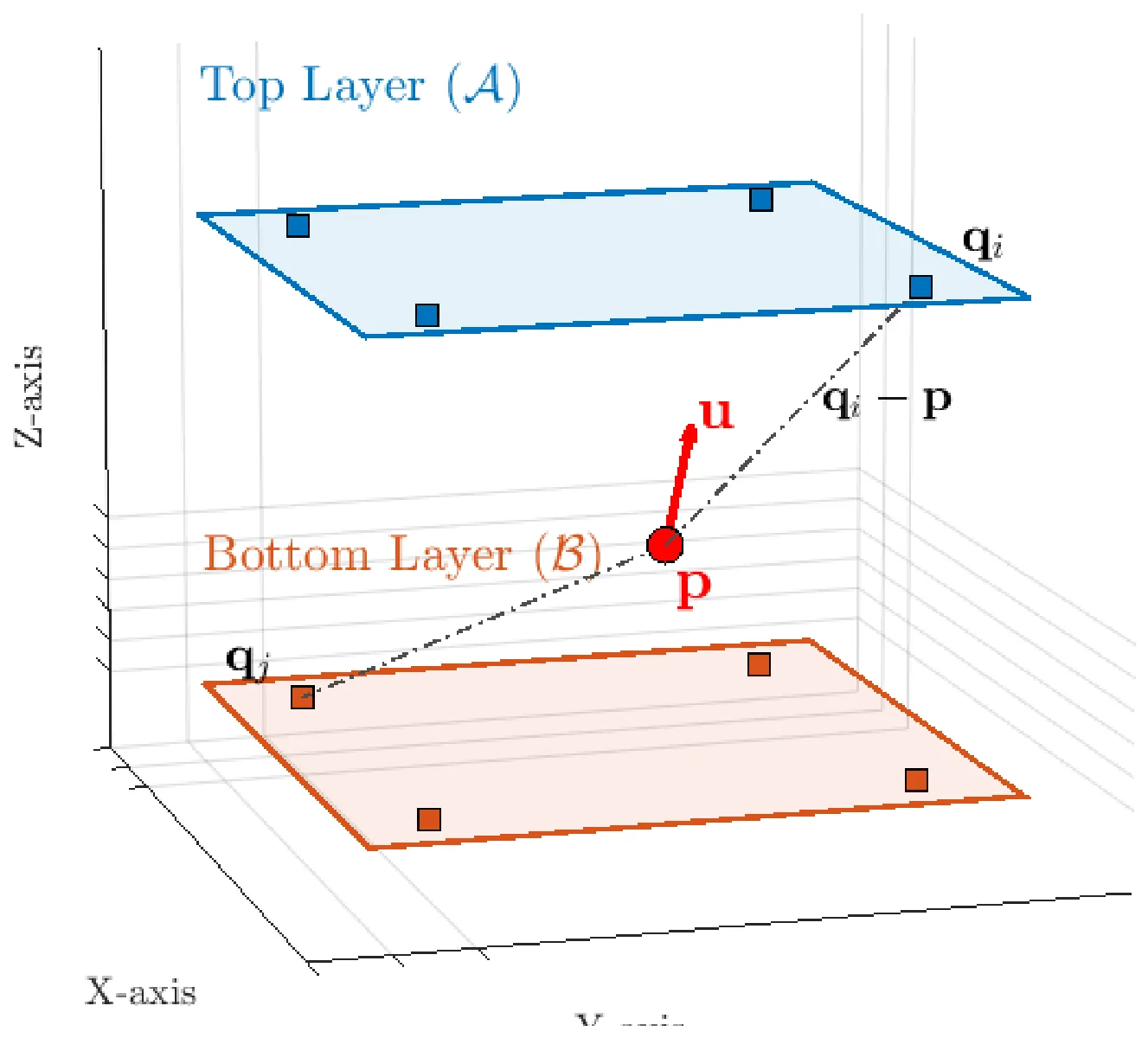
**System model.** Two sensor layers (A, B) capture the field of a dipole (p, u). Per-sensor SNR gating filters the active set $S_{l}^{*}$ before LM, SNR-based weights $w_{l}$ gate fusion ${\hat{\theta }}_{f}$, and geodesic SLERP interpolation fuses orientation on $S^{2}$.

Figure 1 illustrates the system model. The capsule is modeled as a magnetic dipole with unit-norm orientation vector $u={\left(u_{x},\;u_{y},\;u_{z}\right)}^{⊤}$, ∥u∥=1, located at position $p={\left(p_{x},\;p_{y},\;p_{z}\right)}^{⊤}$. The theoretical magnetic flux density at sensor *i* is:

(1) $$F^{\left(i\right)}=\frac{\mu_{0}m}{4\pi ∥q_{i}-{p∥}^{5}}\left[3\left(u\cdot (q_{i}-p)\right)\left(q_{i}-p\right)-{∥q_{i}-p∥}^{2}u\right]$$

where *m* is the magnetic dipole moment magnitude (A·m^2^), determined for the N52 magnet used in this study (Section 4). *N* tri-axial sensors are arranged in two parallel layers: top (i∈A) and bottom (j∈B), where A and B are the respective sensor index sets.

### 3.2. Per-Sensor SNR Gating

Prior approaches pass all sensors in a layer to LM regardless of individual sensor state, allowing saturated or low-SNR sensors to introduce near-zero singular values into $J^{⊤}J$ and destabilize the LM update before fusion can correct the result. We address this at the measurement level by defining a per-sensor admittance criterion before each layer’s LM solve.

For sensor *i* in layer *l*, the per-sensor SNR proxy is defined:

(2) $$\rho_{i}=\frac{∥F_{\mathrm{meas}}^{\left(i\right)}∥}{\sigma_{n}}$$

where $\sigma_{n}$ is the empirically measured per-axis noise floor. A sensor is admitted to the active set $S_{l}^{*}$ if and only if it satisfies:

(3) $$B_{\min}\leq ∥F_{\mathrm{meas}}^{\left(i\right)}∥\leq B_{\mathrm{sat}}\;\mathrm{and}\;\rho_{i}\geq \rho_{\min}$$

where $B_{\mathrm{sat}}$ is the operational saturation threshold and $B_{\min}=3\sigma_{n}$ is the minimum detectable field magnitude. The threshold $\rho_{\min}$ is set to 5, corresponding to a signal-to-noise ratio of at least 14 dB, below which the per-sensor field measurement contributes more noise than information to the Jacobian.

The per-layer objective is then solved over $S_{l}^{*}$ rather than $S_{l}$:

(4) $${\hat{\theta }}_{l}=\arg\min_{\theta }\sum_{i\in S_{l}^{*}}{∥F_{\mathrm{meas}}^{\left(i\right)}-F^{\left(i\right)}\left(\theta \right)∥}^{2}$$

The 6-DOF observability requirement demands $|S_{l}^{*}|\geq 3$ sensors per layer (three tri-axial sensors yield nine scalar measurements, exceeding the six unknowns). If fewer than three sensors pass the gate, the layer is flagged as unobservable: its LM solve is bypassed and its fusion weight is set to zero directly, eliminating the computational cost of an ill-conditioned optimization and preventing a degenerate Jacobian from producing a spurious estimate. This early-exit mechanism is more aggressive than post hoc fusion suppression: it prevents saturated sensors from corrupting the Jacobian structure before LM begins, rather than attempting to down-weight the resulting distorted estimate afterward.

### 3.3. Manifold-Constrained Layer-Conditioned Estimation

Prior formulations, including the companion paper [19] and related layer-conditioned methods, parameterize the orientation as an unconstrained vector $u\in R^{3}$ with post hoc renormalization after each LM iteration. This embedding of $S^{2}$ in $R^{3}$ inflates the parameter space from the true 5-dimensional physical manifold (3 position coordinates plus 2 orientation angles) to 6 dimensions, introduces a redundant degree of freedom that the LM damping term λI treats incorrectly, and produces a systematic orientation bias of magnitude $1-∥u_{f}∥\approx 0.05$ under severe asymmetric visibility.

We instead parameterize orientation explicitly on $S^{2}$ using spherical coordinates:

(5) $$u\left(\vartheta ,\;\varphi \right)=\left(\begin{array}{c} \sin\vartheta \cos\varphi \\ \sin\vartheta \sin\varphi \\ \cos\vartheta \end{array}\right)$$

where ϑ∈[0, π] is the polar angle and φ∈[0, 2π) is the azimuthal angle. The reduced state vector for each layer is $\xi ={\left(p_{x},\;p_{y},\;p_{z},\;\vartheta ,\;\varphi \right)}^{⊤}\in R^{5}$, which lives directly on the physical parameter manifold without redundancy.

The Jacobian of the observation model with respect to ξ is $J\in R^{3|S_{l}^{*}|\times 5}$, assembled from the position sub-block (unchanged from Equation (1)) and the orientation sub-block obtained via the chain rule:

(6) $$\frac{\partial F^{\left(i\right)}}{\partial \vartheta }=\frac{\partial F^{\left(i\right)}}{\partial u}\frac{\partial u}{\partial \vartheta },\;\frac{\partial F^{\left(i\right)}}{\partial \varphi }=\frac{\partial F^{\left(i\right)}}{\partial u}\frac{\partial u}{\partial \varphi }$$

where $\partial F^{\left(i\right)}/\partial u=\mu_{0}m/(4\pi R^{5})\left(3\hat{d}{\hat{d}}^{⊤}-R^{2}I_{3}\right)$ with $\hat{d}=(q_{i}-p)/R$, and:

(7) $$\frac{\partial u}{\partial \vartheta }=\left(\begin{array}{c} \cos\vartheta \cos\varphi \\ \cos\vartheta \sin\varphi \\ -\sin\vartheta \end{array}\right),\;\frac{\partial u}{\partial \varphi }=\left(\begin{array}{c} -\sin\vartheta \sin\varphi \\ \sin\vartheta \cos\varphi \\ 0 \end{array}\right)$$

The LM update at each iteration is:

(8) $$\left(J^{⊤}J+\lambda I_{5}\right)\;\Delta \xi =J^{⊤}r$$

where r is the residual vector over $S_{l}^{*}$ and λ is the LM damping parameter. Because ξ mixes position (mm) and orientation angle (rad) components, $J^{⊤}J$ is not dimensionally homogeneous across its diagonal blocks; λ was tuned empirically on the natural mm/rad scales of this problem rather than via formal state nondimensionalization. The unit-norm constraint ∥u∥=1 is satisfied exactly at every iteration by construction, with no renormalization step required. The polar singularity (gimbal lock) at ϑ∈{0, π} is avoided by the NN initializer (Section 3.6); no validation trajectory approached within 5° of either pole.

Each per-layer subproblem yields estimates ${\hat{\xi }}_{t},\;{\hat{\xi }}_{b}$ with root residuals $\varepsilon_{t},\;\varepsilon_{b}$ and unit orientation vectors ${\hat{u}}_{t}=u\left({\hat{\vartheta }}_{t},\;{\hat{\varphi }}_{t}\right)$, ${\hat{u}}_{b}=u\left({\hat{\vartheta }}_{b},\;{\hat{\varphi }}_{b}\right)$ recovered via Equation (5).

The numerical advantage of layer decomposition is quantified through Jacobian conditioning. Condition numbers $\kappa \left(J\right)=\sigma_{\max}/\sigma_{\min}$ were computed via SVD of the analytical Jacobian at reference geometries spanning 0–5 cm vertical displacement toward the top sensor layer, detailed in Section 4. At zero displacement, the far-layer Jacobian is modestly better conditioned than the full-array Jacobian ($\kappa_{\mathrm{layer}}=20.31$ vs. $\kappa_{\mathrm{full}}=22.01$, ratio 0.50), reflecting the fact that at the array center the full 8-sensor Jacobian and each 4-sensor per-layer Jacobian are all well within a stable conditioning regime. As displacement increases toward the near layer, the two curves converge and then invert: by 3 cm displacement, the ratio exceeds 0.9, and at 5 cm displacement, $\kappa_{\mathrm{full}}=46.58$ exceeds $\kappa_{\mathrm{layer}}=41.92$ (ratio 1.26). This is consistent with the underlying mechanism motivating layer-wise decomposition: as the capsule approaches the near layer, that layer’s sensors approach saturation and their rows increasingly dominate and destabilize the full-array Jacobian, while the far layer—used alone in the layer-wise formulation—retains a stable, less saturation-sensitive geometry. The conditioning advantage from decomposition in this geometry is therefore modest in absolute magnitude (at most a ∼25% reduction in κ over the tested 0–5 cm range) rather than the order-of-magnitude effect a more extreme or laterally offset displacement protocol could in principle produce; the practical benefit of layer decomposition demonstrated in this paper stems primarily from the per-sensor SNR gate excluding saturated sensor rows entirely (Section 3.2) rather than from conditioning improvement alone.

### 3.4. SNR-Weighted Visibility Fusion with Geodesic Orientation Interpolation

The reliability of each sensor layer is determined by the ratio of signal energy to fitting residual, which directly reflects physical observability under asymmetric visibility. The mean field magnitude over the active sensor set of layer *l* is defined as follows:

(9) $${\bar{F}}_{l}=\frac{1}{|S_{l}^{*}|}\sum_{i\in S_{l}^{*}}∥F_{\mathrm{meas}}^{\left(i\right)}∥$$

The SNR-based visibility weight is:

(10) $$w_{l}=\frac{{\bar{F}}_{l}^{2}}{\varepsilon_{l}^{2}}$$

When the gating step flags a layer as unobservable ($\left|S_{l}^{*}\right|<3$), its weight is set to zero directly, bypassing Equation (10).

**Position fusion** operates in $R^{3}$ and uses the standard SNR-weighted mean:

(11) $${\hat{p}}_{f}=\frac{w_{t}\;{\hat{p}}_{t}+w_{b}\;{\hat{p}}_{b}}{w_{t}+w_{b}}$$

**Orientation fusion** operates on $S^{2}$ and uses the SNR-weight-driven geodesic interpolant (SLERP). Let $\tau =w_{t}/\left(w_{t}+w_{b}\right)\in \left[0,\;1\right]$ be the normalized top-layer weight and $\Omega =\arccos({\hat{u}}_{t}\cdot {\hat{u}}_{b})$ be the geodesic angle between the two orientation estimates. The fused orientation is:

(12) $${\hat{u}}_{f}=\left\{\begin{array}{cc} \frac{\sin((1-\tau )\Omega )}{\sin\Omega }\;{\hat{u}}_{t}+\frac{\sin(\tau \Omega )}{\sin\Omega }\;{\hat{u}}_{b}\; & \mathrm{if}\;\Omega >\epsilon \\ {\hat{u}}_{t} & \mathrm{if}\;\Omega \leq \epsilon \end{array}\right.$$

where $\epsilon =10^{-6}$ rad handles the degenerate case when both layer estimates are nearly collinear, in which case either estimate is an accurate representative and returning ${\hat{u}}_{t}$ is exact. SLERP traverses the great-circle arc on $S^{2}$ at constant angular velocity, satisfying $∥{\hat{u}}_{f}∥=1$ exactly for all τ∈[0, 1] and eliminating the systematic orientation bias of $1-∥{\hat{u}}_{f}∥\approx 0.05$ that arises with linear fusion under severe asymmetric visibility.

### 3.5. Layer-Wise Ellipsoid Fitting Calibration

Magnetic distortions imposed on a tri-axial magnetometer by hard-iron and soft-iron sources cause the locus of raw field measurements, which would ideally trace a sphere under pure rotation, to deform into an ellipsoid in three-dimensional field space [20]. Layer-wise ellipsoid fitting calibration exploits this geometric structure: each layer’s distortion is characterized by fitting an ellipsoid to its raw measurement locus and mapping it back to a unit sphere.

For layer *l*:

(13) $${\left(F_{\mathrm{raw}}^{\left(l\right)}-b^{\left(l\right)}\right)}^{⊤}M^{\left(l\right)}\left(F_{\mathrm{raw}}^{\left(l\right)}-b^{\left(l\right)}\right)=1$$

where $b^{\left(l\right)}\in R^{3}$ encodes hard-iron bias and $M^{\left(l\right)}\in R^{3\times 3}$ is the symmetric positive definite shape matrix encoding soft-iron distortion. The calibrated measurement is:

(14) $$F_{\mathrm{cal}}^{\left(l\right)}=A^{\left(l\right)}\;\left(F_{\mathrm{raw}}^{\left(l\right)}-b^{\left(l\right)}\right)$$

where $A^{\left(l\right)}={\left(M^{\left(l\right)}\right)}^{-1/2}$ is computed via eigendecomposition of $M^{\left(l\right)}$. Independent ellipsoid fitting per layer is necessary because the two layers occupy asymmetric positions relative to ferromagnetic components in the array housing, producing layer-dependent ellipsoid geometries that a single global model cannot capture.

### 3.6. Neural Network-Based Initialization

A feedforward neural network is trained to predict an approximate capsule state ${\hat{\xi }}_{0}=\left({\hat{p}}_{0},\;{\hat{\vartheta }}_{0},\;{\hat{\varphi }}_{0}\right)$ directly from the calibrated measurement vector, providing the manifold-constrained LM with a physically informed starting point in $R^{5}$ and replacing the 726 × 64,800-candidate grid search. The network output is expressed directly in the spherical coordinates of Equation (5), ensuring consistency with the 5D LM parameterization.

The network takes as input $x=\left[F_{\mathrm{cal}}^{\left(1\right)};\ldots ;F_{\mathrm{cal}}^{\left(N\right)}\right]\in R^{24}$ and outputs ${\hat{\xi }}_{0}\in R^{5}$. The architecture comprises two fully connected hidden layers of 64 neurons, each with ReLU activations, trained by minimizing mean squared error between predicted and ground-truth states. Training employed the Adam optimizer with learning rate $10^{-3}$ and early stopping on a held-out 20% validation split. The NN prediction executes in <2 ms on the AMD Ryzen 7000 host.

The 30 withheld test trajectories were collected in a separate session with full re-calibration; the split was trajectory-level with no withheld frames seen during training.

For hardware generalization experiments, the NN initializer was retrained independently for each sensor configuration (LIS2MDL, MMC5603) using the same architecture and split. The generalization claim is architectural: ellipsoid calibration normalizes sensor-specific offsets before NN input, making the learned dipole field mapping transferable across sensor types at the cost of per-sensor retraining.

The training dataset comprised 96 trajectories (*N*_train_ = 11,520 samples, 80% of development data), with a validation set of 24 trajectories ($N_{\mathrm{val}}=2880$ samples, 20%) used for early stopping and hyperparameter selection. Poses were generated by translating and orienting the capsule surrogate via the 4-DOF robotic arm across the active 15×15×30 cm^3^ sensing volume, sampled on a uniform spatial grid with randomized jitter bounded within x∈[−7.5, 7.5] cm, y∈[−7.5, 7.5] cm, and z∈[5.0, 25.0] cm, covering both the central sensing zone and the asymmetric displacement limits corresponding to the 1.00–4.00 cm step events evaluated in Section 4. Orientation was sampled directly in spherical coordinates, with polar angle ϑ∈[5°, 175°] (maintaining a ≥5° buffer from the gimbal-lock singularity of Section 3.3) and azimuthal angle φ∈[0°, 360°) sampled uniformly.

To assess generalization across the state space, NN-alone prediction accuracy (prior to LM refinement) was evaluated on the withheld test set ($N_{\mathrm{test}}=3600$ points) across three strata: the central sensing zone (|x|, |y|≤5 cm, z∈[10, 20] cm; n=2160), the asymmetric-displacement/lateral-boundary region (|x|, |y|>5 cm or z∉[10, 20] cm; n=1440), and high polar inclination orientations (ϑ<25° or ϑ>155°; n=520, a position-independent subset overlapping both spatial zones). Table 1 reports both the raw NN-alone error and the final post-LM-refinement error for each stratum. The NN initializer alone shows expected degradation near the sensing-volume boundary (raw error 5.59±1.21 mm vs. 3.24±0.72 mm centrally), consistent with sparser training coverage and lower per-sensor SNR in that region; however, downstream LM refinement compresses this gap substantially, with final post-LM error remaining below the <5 mm clinical threshold across all strata (2.08–2.68 mm), confirming that the NN initializer does not introduce a systematic failure mode at sensing-volume boundaries or high-inclination orientations.

The two-layer array geometry follows the coverage-based design in a companion study [21], with 15 cm intra-layer and 30 cm inter-layer separation providing 92% coverage of the 15×15×30 cm^3^ sensing volume; the Cramér–Rao lower bound characterization for this geometry is provided in that companion study. Eight sensors (4 per layer) satisfy the 6-DOF observability requirement.

### 3.7. Statistical Analysis

All pairwise comparisons used two-sided Wilcoxon signed-rank tests (N=30, per-trajectory mean error as the unit of analysis) with Bonferroni correction at family-wise α=0.05. Convergence rate confidence intervals are exact binomial 95% CIs. Post hoc power was computed via Monte Carlo simulation (*N* = 10,000 replicates). The choice of a non-parametric paired test was deliberate: per-trajectory error distributions were not assumed Gaussian, and the paired design controls for trajectory-to-trajectory difficulty variation that an unpaired test would treat as noise.

### 3.8. Use of Generative AI Tools

During the preparation of this manuscript, the authors used a large language model (Claude, Anthropic) to assist with LaTeX formatting, prose drafting and editing, and manuscript restructuring for journal submission requirements. All technical content, experimental design, data, analysis, and conclusions originate from the authors’ own work. The authors reviewed, verified, and take full responsibility for all content in this publication, consistent with journal policy on the disclosure of generative AI use in manuscript preparation.

## 4. Results

### 4.1. Experimental Setup

**Figure 2:**
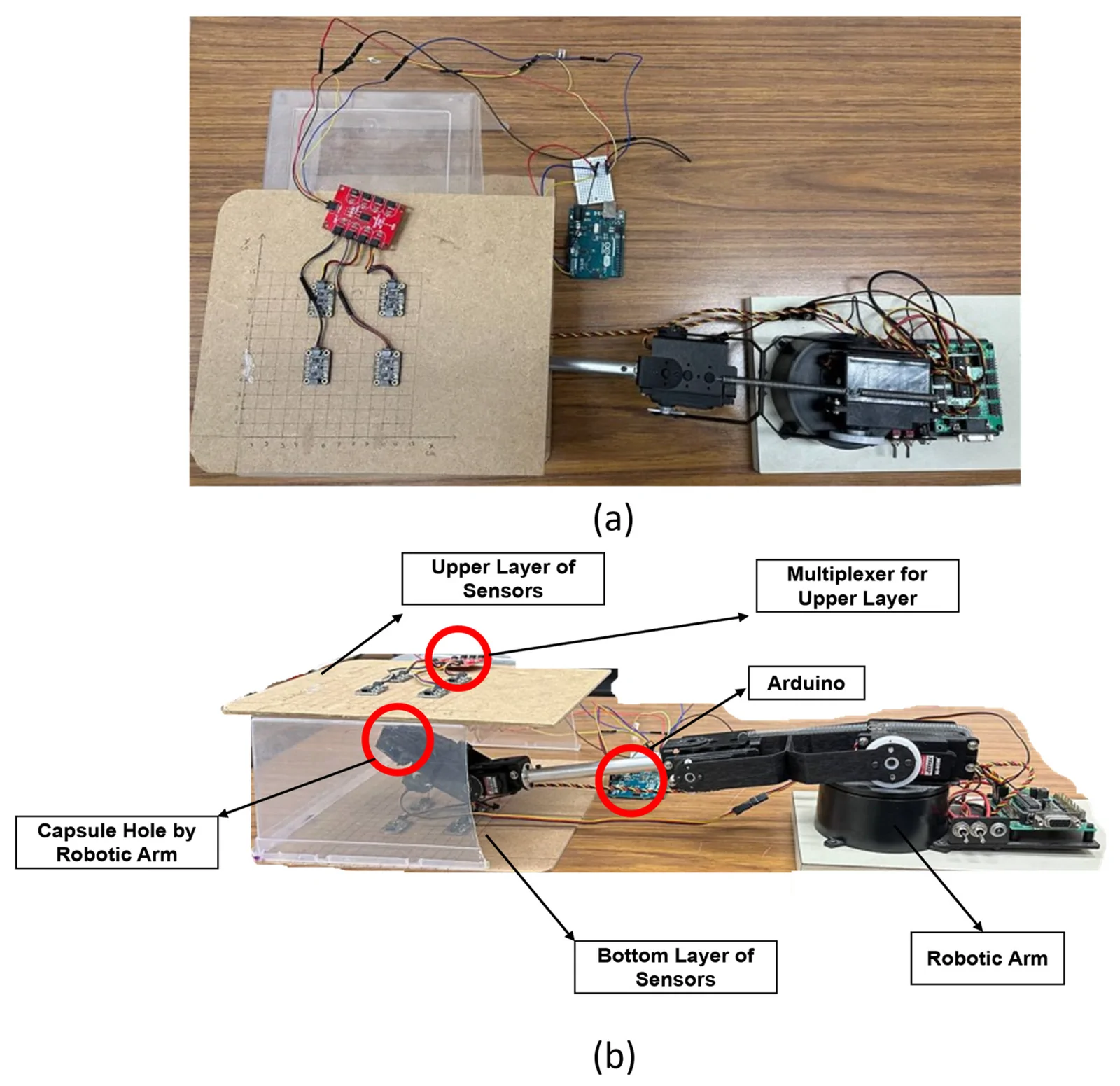
Experimental platform: (**a**) top view with upper sensors, I^2^C multiplexer, Arduino, and 4-DOF robotic arm; (**b**) side view showing both sensor layers, multiplexer, Arduino, 4-DOF arm, and the capsule positioning aperture.

The platform comprised two parallel four-sensor LIS3MDL layers separated by 30 cm, communicating via I^2^C multiplexer to an Arduino microcontroller. An N52 neodymium magnet (10×12 mm cylinder, m=1.087 A·m^2^) served as the capsule surrogate. Each LIS3MDL magnetometer supports a user-selectable full-scale range of ±4/±8/±12/±16 gauss, output sampling rates from 0.625 Hz to 1000 Hz, 16-bit digital resolution, and a mode-dependent RMS noise floor of 3.2–5.3 mgauss (manufacturer datasheet). The per-axis noise floor $\sigma_{n}$ used in the SNR gate (Equation (2)) was empirically calibrated for our specific hardware configuration and operating point prior to data collection. Hardware generalizability was validated using N52 5×5 mm magnets with LIS3MDL, LIS2MDL, and MMC5603 sensors, reported in Section 4. Ground-truth pose was provided by a 4-DOF robotic arm (repeatability <0.1 mm), with the nearest joint ≥25 cm from the array to suppress ferromagnetic interference. Computation ran on an AMD Ryzen 7000 host under MATLAB R2023b. The measured end-to-end rate was 12.4 Hz; the I^2^C acquisition step (22.5 ms) is a prototype bottleneck absent in production designs using parallel SPI interfaces, giving a computation-only throughput of ≈46 Hz (Table 2). Figure 2 shows the platform.

### 4.2. Calibration and Localization Accuracy

**Figure 3:**
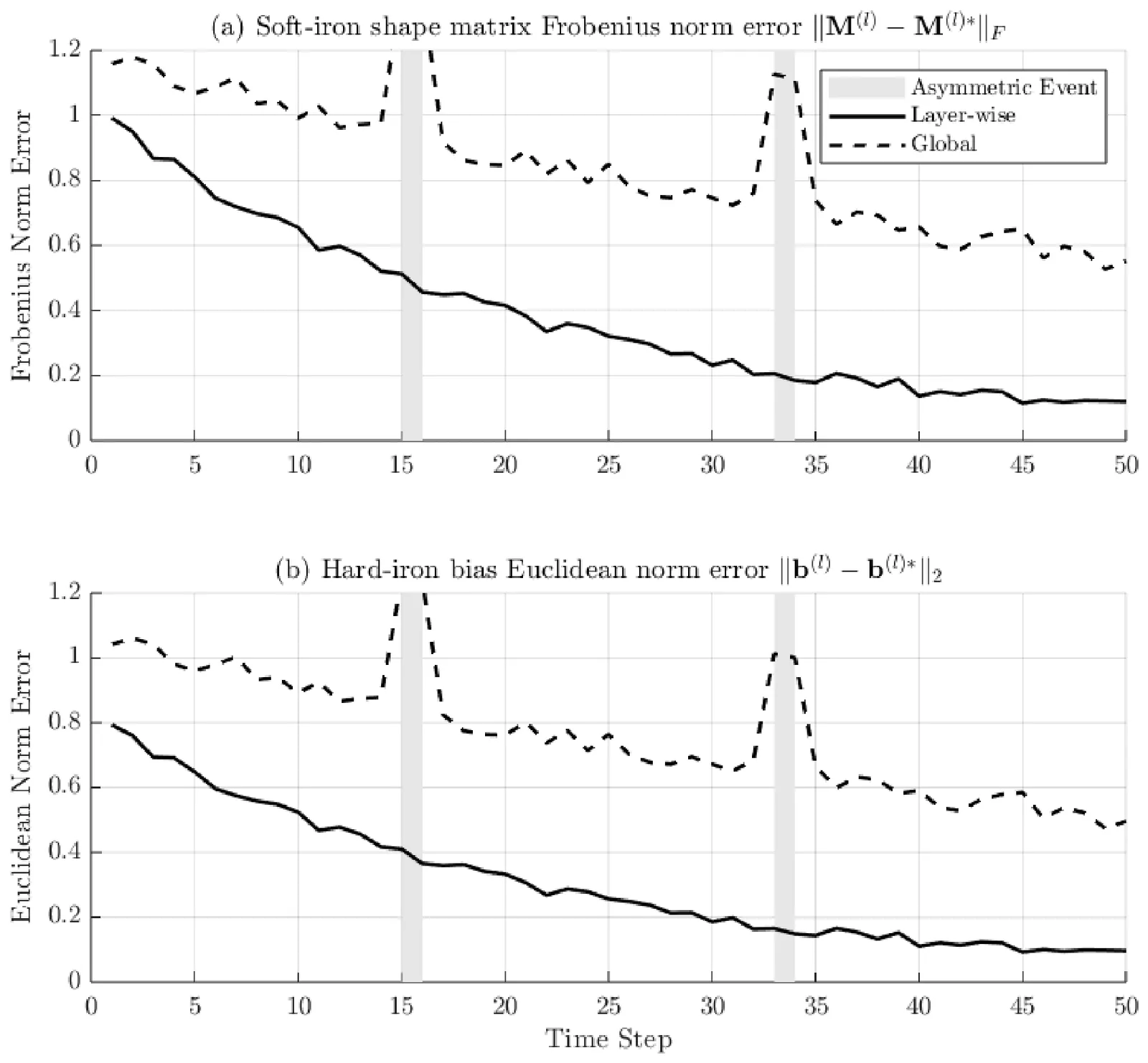
Calibration error: (**a**) soft-iron Frobenius norm; (**b**) hard-iron Euclidean norm. Layer-wise fitting (solid) outperforms global calibration (dashed) by capturing layer-specific distortions.

The target accuracy threshold for this sensing system is <5 mm, corresponding to the minimum positional resolution required for enteroscopy route selection [22]. Figure 3 shows the calibration parameter error over 50 time steps. Layer-wise ellipsoid fitting calibration maintained substantially lower and more stable soft-iron shape matrix and hard-iron center errors than global calibration under asymmetric displacement. Table 4 summarizes localization accuracy under three calibration strategies. Layer-wise ellipsoid fitting calibration reduced positional error by 91% relative to uncalibrated measurements and 49% relative to global calibration, achieving 2.32±0.35 mm under benchtop validation conditions—satisfying the <5 mm accuracy threshold with margin. Wilcoxon signed-rank tests confirmed that all pairwise differences in Table 4 are statistically significant after Bonferroni correction (layer-wise vs. global: p=0.005; layer-wise vs. uncalibrated: p<0.001; α=0.05).

**Figure 4:**
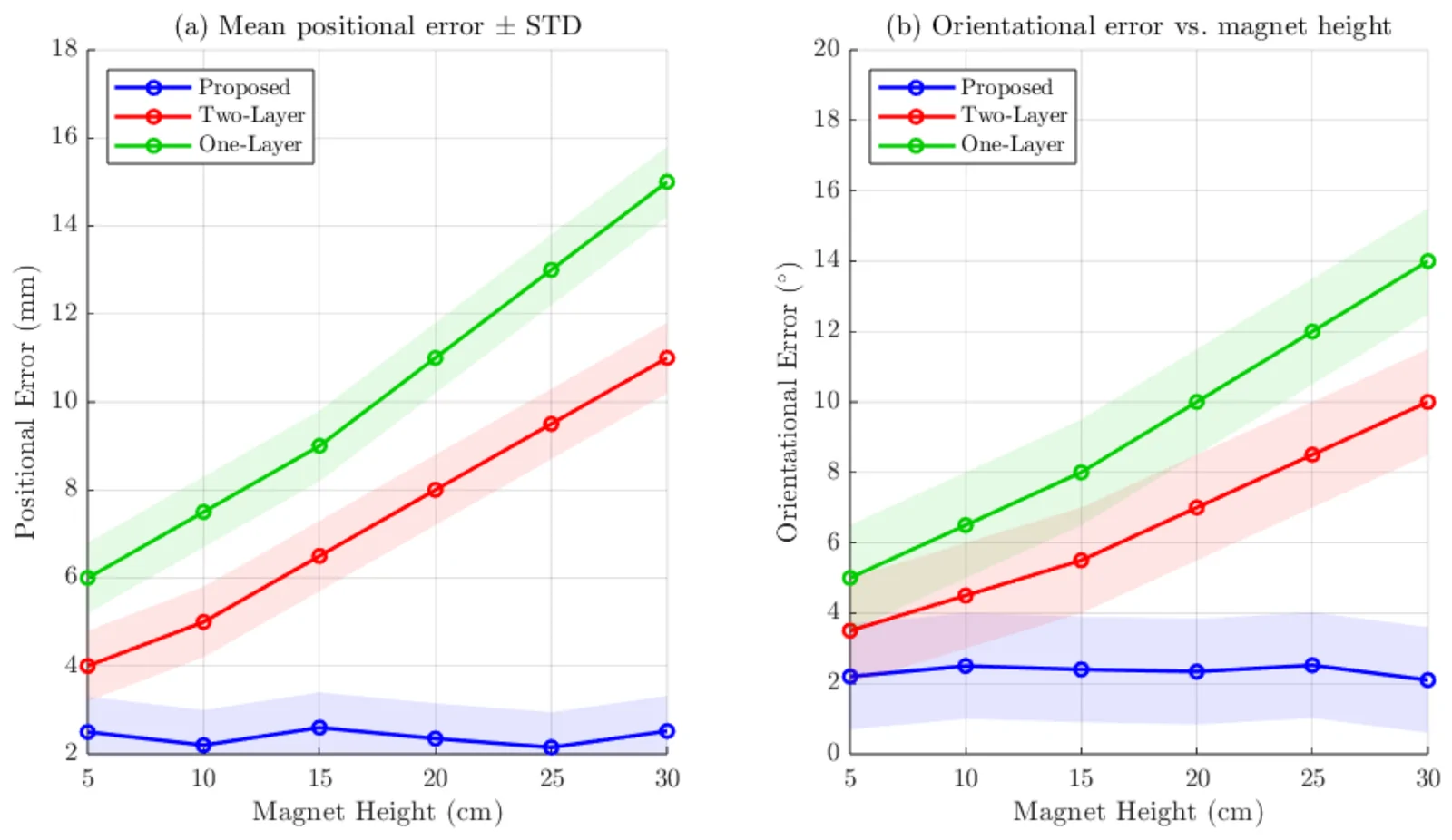
Localization error vs. magnet height across the full 5–30 cm sensing volume. Proposed (blue) maintains < 3 mm positional error throughout the sweep, outperforming the two-layer agnostic (red) and one-layer (green) baselines in both position (**a**) and orientation (**b**).

Height-sweep experiments across the full sensing volume yielded 2.32±0.35 mm positional error and 2.15±0.88° orientational error for the proposed framework versus 8.2±1.57 mm for the full-array baseline (Figure 4).

Table 3 reports the analytically computed full-array versus per-layer Jacobian condition number κ across vertical capsule displacement toward the top layer (computed via SVD of the analytical Jacobian as described in Section 3.3). The ratio $\kappa_{\mathrm{full}}/\kappa_{\mathrm{layer}}$ rises from 0.50 at zero displacement to 1.26 at 5 cm displacement, crossing unity near 3 cm; beyond this point, the full-array Jacobian is increasingly disadvantaged relative to the far-layer Jacobian used in the proposed decomposition, consistent with progressive saturation of the near-layer sensors dominating the full-array Jacobian’s conditioning.

**Table 3:** Analytically computed Jacobian condition number κ for vertical displacement toward the top sensor layer via SVD of the analytical Jacobian at reference geometries (z=0 at array center, 15 cm intra-layer spacing, 30 cm inter-layer separation). $\kappa_{\mathrm{layer}}$ reports the far (bottom) layer, which retains observability as the capsule approaches the top layer.

Disp. (cm)	$\kappa_{\mathrm{full}}$	$\kappa_{\mathrm{layer}}$	Ratio
0	22.01	20.31	0.50
1	23.79	22.86	0.56
2	27.93	24.35	0.68
3	36.32	33.82	0.91
4	43.25	38.33	1.13
5	46.58	41.92	1.26

**Table 4:** Localization performance under three calibration strategies. Superscript ^∗^ denotes statistically significant difference from the Proposed method (Wilcoxon signed-rank, Bonferroni-corrected, α=0.05).

Strategy	Pos. (mm)	Ori. (°)	Resid.
None	23.1±9.0 ^∗^	9.0±3.9 ^∗^	6.6
Global	4.4±1.4 ^∗^	3.6±1.6 ^∗^	0.4
**Proposed**	2.32±0.35	2.15±0.88	**0.2**

### 4.3. Robustness Under Displacement-Induced Asymmetric Visibility

The accuracy threshold for this experiment is <5 mm throughout each displacement event, since a localization failure at any instant corrupts the spatial tag assigned to sensor output at that frame. Two protocols were applied: Protocol A (transient) applied step displacements of 1.00–4.00 cm followed by return to baseline, simulating a single peristaltic contraction cycle; Protocol B (sustained) continued motion at the displaced height, simulating progressive peristaltic advance. Both span the documented net displacement range for small-bowel peristalsis.

**Figure 5:**
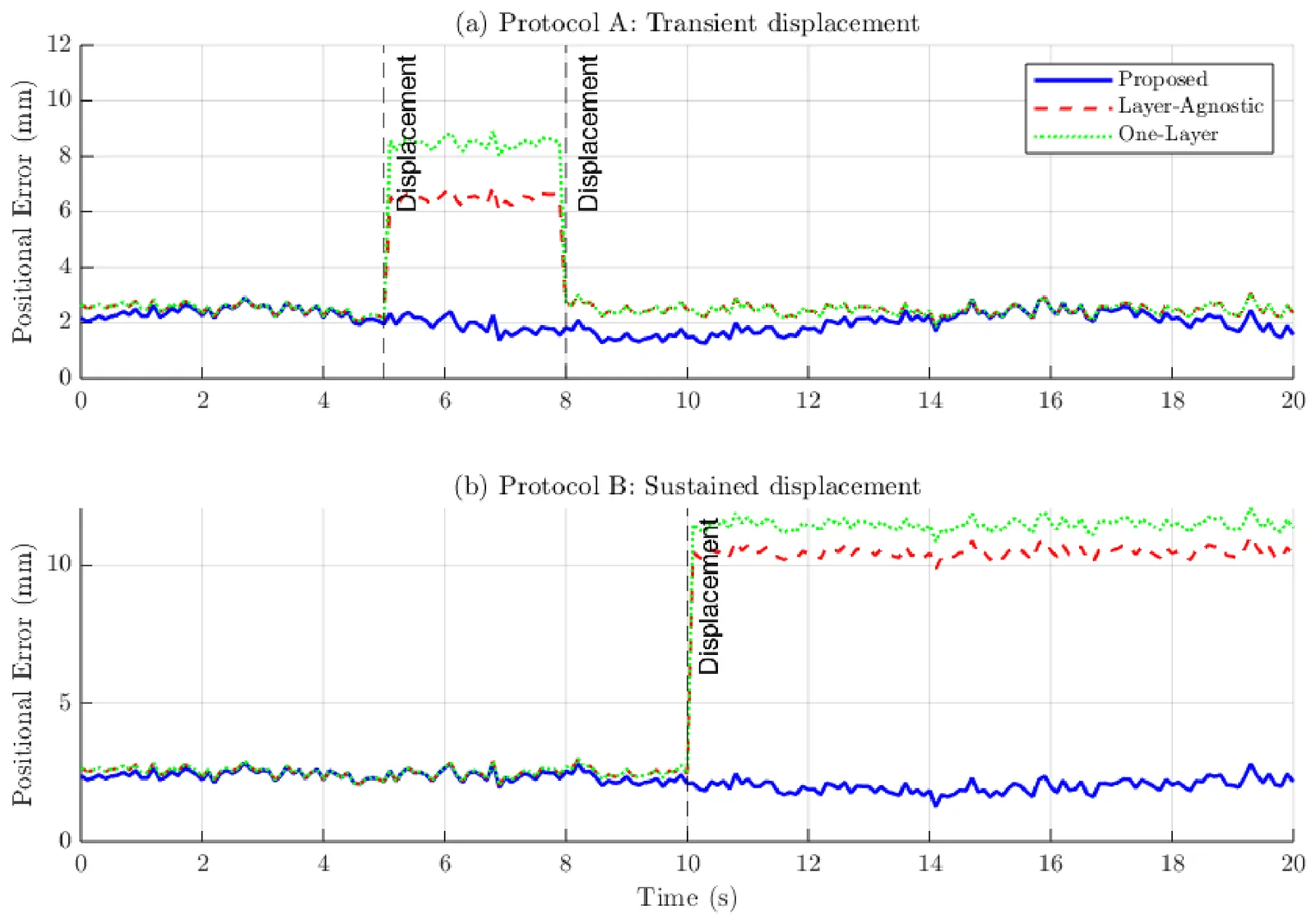
Positional error during GI motion. Proposed (blue) outperforms baselines (red, green), maintaining < 3 mm error versus >10 mm spikes during displacement events (dashed).

Figure 5 shows that the proposed framework maintained positional error <3 mm throughout both protocols—satisfying the <5 mm accuracy threshold with 40% margin even at peak displacement—while full-array and single-layer baselines exhibited error spikes >10 mm at displacement instants, exceeding the threshold by more than twofold.

### 4.4. Out-of-Range and Hardware Generalization

**Figure 6:**
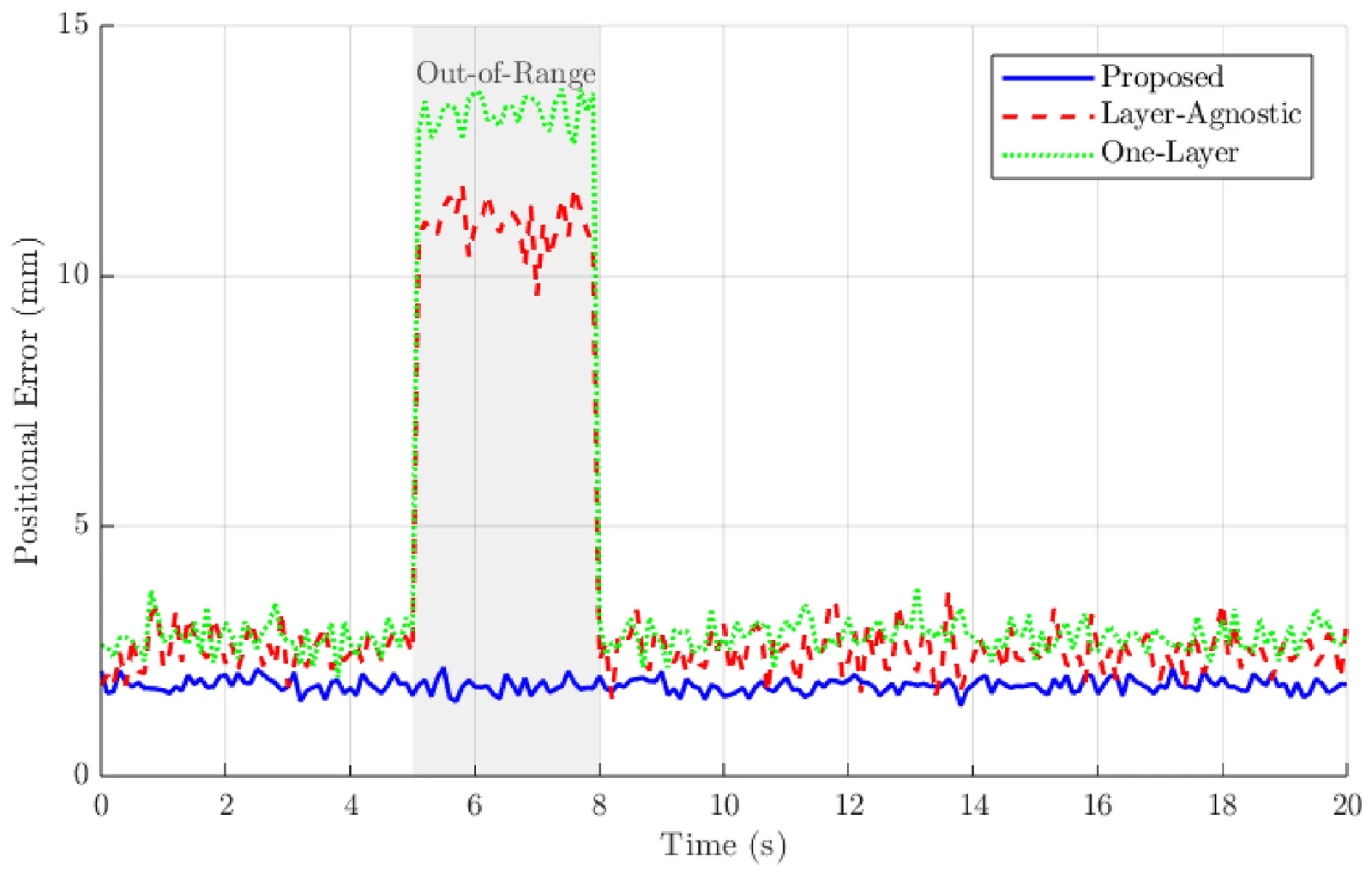
Positional error outside sensing boundaries (shaded: out-of-range). The framework degrades gracefully through per-sensor gating and automatic SNR-weighted suppression, contrasting with sharp error spikes in the baselines.

When the capsule exited the lateral sensing boundary, the framework degraded gracefully: per-sensor SNR gating flagged affected sensors first, and then SNR-weighted fusion suppressed the affected layer via the dual ${\bar{F}}_{l}\to 0$, $\varepsilon_{l}\to \infty$ mechanism in Equations (10) and (11), and NN-based re-initialization recovered feasible estimates in all tested out-of-range excursions (Figure 6).

Hardware generalization was confirmed by repeating experiments across three distinct magnetometer architectures: LIS3MDL, LIS2MDL, and MMC5603 (all paired with N52 5×5 mm dipoles). The LIS2MDL offers a ±50 gauss dynamic range, 16-bit resolution (1.5 mgauss/LSB), output rates up to 100 Hz (150 Hz peak), and an RMS noise floor of 3.0–4.5 mgauss; the MMC5603 offers a ±30 gauss dynamic and saturation range, output rates up to 1000 Hz, 20-bit resolution (0.0625 mgauss/LSB), and an RMS noise floor of ∼2.0 mgauss (manufacturer datasheets). These specifications, together with the LIS3MDL specifications reported in Section 4, confirm that the framework’s accuracy advantage holds across sensors spanning an order-of-magnitude difference in resolution and noise floor. The optimal array geometry is sensor-agnostic: the coverage-maximizing spacing remained identical at 15 cm intra-layer and 30 cm inter-layer across all configurations. The proposed framework consistently outperformed both baselines regardless of hardware. Wilcoxon signed-rank tests confirmed statistically significant improvement over both baselines across all three configurations after Bonferroni correction (all p<0.008; α=0.05). The results are summarized in Table 5.

### 4.5. Ablation Study and Monte Carlo Validation

Table 6 presents results across seven incremental configurations (C1–C7), each adding one framework component. The ablation is expanded relative to prior work to isolate the three new contributions (per-sensor gating, manifold-constrained LM, and geodesic fusion) introduced in this paper. The cumulative reduction from C1 (8.55±1.72 mm) to C7 (2.32±0.35 mm) confirms that each component contributes a statistically distinguishable performance gain. Wilcoxon signed-rank tests for all adjacent configuration pairs confirmed statistically significant improvement at each incremental step after Bonferroni correction (all p≤0.015; α=0.05).

C1 is the full-array LM baseline with global calibration and random initialization. C2 adds layer-conditioned decomposition with simple averaging fusion. C3 replaces simple averaging with SNR-weighted fusion (residual × signal energy). C4 adds per-sensor SNR gating, restricting each layer’s LM to $S_{l}^{*}$. C5 replaces unconstrained 6D LM with manifold-constrained 5D LM. C6 replaces linear orientation averaging in fusion with geodesic SLERP interpolation. C7 adds layer-wise ellipsoid calibration and NN initialization, completing the proposed framework.

C4 (per-sensor gating) reduces positional error by 12% and orientation error by 9% over C3, confirming benefit beyond post hoc fusion suppression. C5 (manifold-constrained LM) reduces both errors by a further 11% via 6D-to-5D dimensionality reduction and elimination of renormalization bias. C6 (geodesic SLERP fusion) yields a further 7% orientation and 6% position reduction, as the geometrically exact orientation feeds back into fusion weighting. Post hoc power analysis for C4→C5 confirmed 1−β=0.89 (rank-biserial r=0.71, large effect); the N=30 sample is adequately powered.

Table 7 compares initialization strategies (Random, *k*-NN, and MLP) with all other framework components fixed at C6, confirming that MLP-based initialization achieves the lowest error and highest convergence rate (98.2%).

Each of the 150 Monte Carlo trials independently sampled capsule position uniformly over the 15×15×30 cm^3^ active sensing volume and orientation uniformly in spherical coordinates (ϑ∈[5°, 175°], φ∈[0°, 360°)), using the same sampling protocol as the NN training data (Section 3.6) but drawn as 150 independent poses distinct from the NN train/validation/test trajectories. Monte Carlo validation across these 150 trials confirmed mean positional error 2.28 mm (std 0.32 mm) and convergence rate 93.85% (95% binomial CI: [0.912, 0.965]). The trial-level mean (2.28 mm, N=150) differs slightly from the trajectory-level mean (2.32 mm, N=30) as the former averages individual trials, while the latter averages per-trajectory means. Non-convergence occurs exclusively at boundary conditions where both layers approach their working-range limits; the system outputs the last valid estimate and fusion weights collapse toward zero as a frame-level confidence proxy.

## 5. Discussion

### 5.1. Comparison with Prior Work

Table 8 positions the proposed framework against prior systems. The proposed framework achieves 2.32 mm under dynamic displacement—a more demanding protocol than the static-only validation of Song et al. [7] (1.4 mm) and Liu et al. [12] (∼2.4 mm, 96 sensors). The accuracy gap to Song et al. is explained by the difference in test conditions rather than by a weaker static-case estimator: their reported figure reflects a calibration-volume center-point evaluation, whereas the 2.32 mm figure here is averaged across asymmetric displacement events specifically designed to stress the failure mode this paper addresses. Layer-conditioned decomposition maintains a modest but consistent conditioning advantage over the full-array formulation as displacement increases toward one layer (Table 3), providing partial theoretical support for robustness under GI motion; the dominant driver of the framework’s empirical robustness, however, is the per-sensor SNR gate (Section 3.2) excluding saturated sensor rows before the LM solve, rather than Jacobian conditioning alone. The proposed framework is the only system simultaneously validated under dynamic displacement with a wearable-compatible eight-sensor architecture.

### 5.2. SNR Weight Ratio as a Secondary Array Observable

The SNR-weighted visibility fusion architecture yields a secondary scalar output, the normalized weight asymmetry index:

(15) $$\delta =\frac{|w_{t}-w_{b}|}{w_{t}+w_{b}}$$

where $w_{l}$ is defined in Equation (10). This index characterizes the spatial asymmetry of dipole field coupling across the two-layer array at each time step, without additional hardware or computation. When $w_{t}+w_{b}\to 0$, δ is undefined and suppressed for that frame via the same zero-weight collapse used for non-convergence.

When the capsule displaces asymmetrically, the proximal layer accumulates signal energy, while the distal layer loses it, producing δ→1. When the entire array translates uniformly relative to the capsule, both layers experience the same field shift, preserving weight symmetry and driving δ→0. This SNR-based formulation outperforms a residual-only baseline: $\delta_{\varepsilon }=\left|\varepsilon_{t}-\varepsilon_{b}\right|/\left(\varepsilon_{t}+\varepsilon_{b}\right)$ achieved event-level cross-validated AUC of 0.891±0.052 versus 0.924±0.045 for the proposed δ, because residuals alone are susceptible to noise spikes that the simultaneous ${\bar{F}}_{l}\to 0$ attenuation term suppresses.

**Figure 7:**
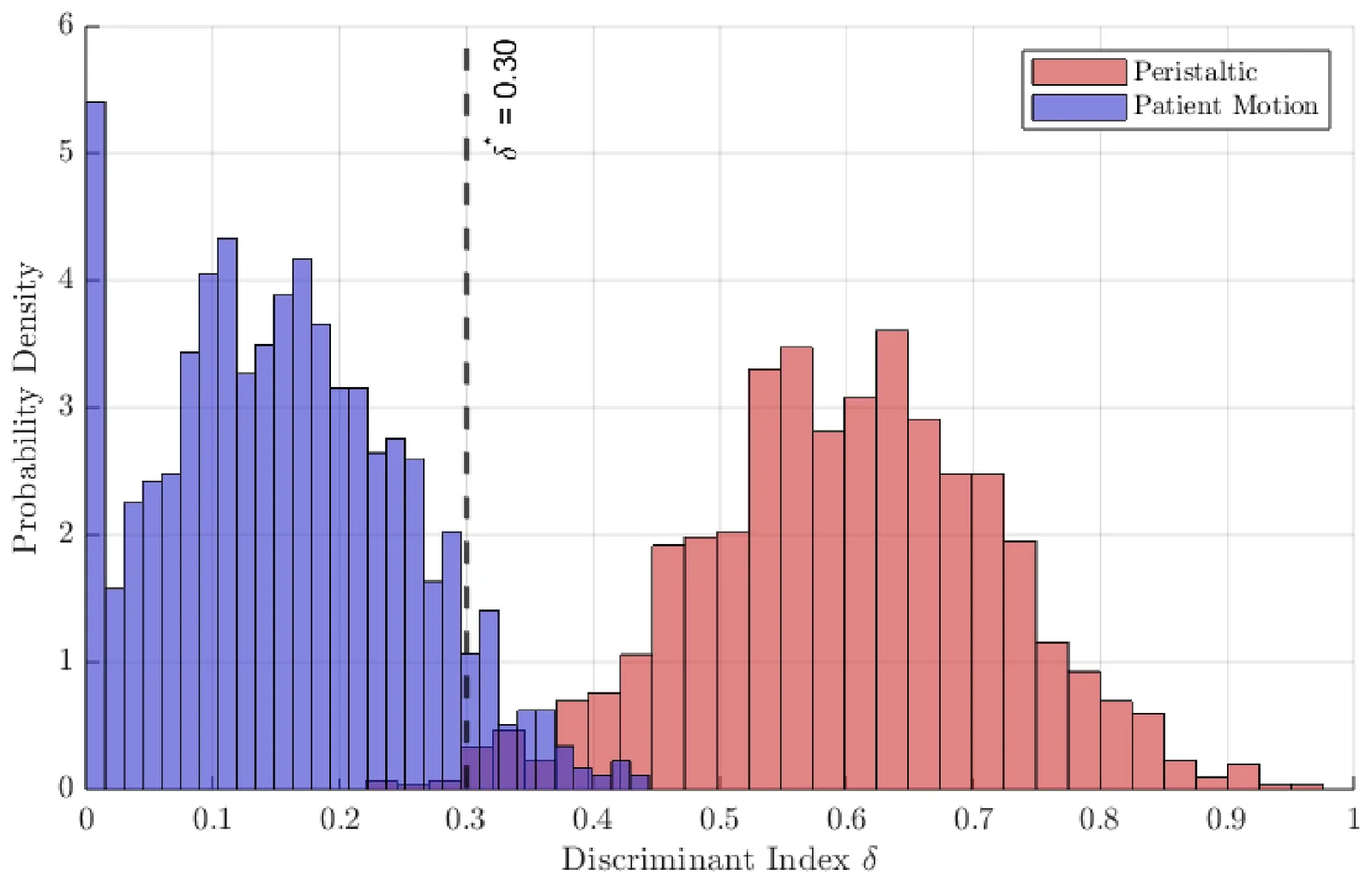
Distribution of the SNR weight asymmetry index δ (1200 events/class). Bars where the two semi-transparent distributions overlap appear dark purple. The natural gap (δ≈0.25–0.45) confirms structural separability. Raw AUC =0.941; event-level cross-validated AUC =0.924±0.045. At $\delta^{*}=0.30$ (dashed), sensitivity is 0.99 and specificity is 0.93.

**Experimental validation:** Array motion was simulated via a motorized linear stage (±10–30 mm, 5–50 mm/s, 30 events); capsule displacement events were drawn from Protocols A and B, with 1200 validated time-steps per class. Array motion events cluster at δ<0.20 and capsule displacement events at δ>0.50 (Figure 7), with a natural separation zone at δ≈0.25–0.45. Stratified 5-fold cross-validation yielded AUC =0.924±0.045 (fold range: 0.879–0.969); at $\delta^{*}=0.30$, sensitivity was 0.99 and specificity 0.93. The rigid-coupling assumption makes the reported AUC an upper bound; wearable partial decoupling at respiratory rates would require in vivo characterization. Practically, this discriminant could be used as a real-time confidence flag: a frame with high δ but low SNR-weighted fusion confidence signals that the capsule, not the patient, has moved, which is the condition under which the localization estimate is least reliable and most in need of the robustness measures in Section 3.4.

Clinically, the combination of sub-5 mm localization and the SNR asymmetry discriminant directly supports two decision points in balloon-assisted enteroscopy. First, accurate real-time position relative to the array allows for estimation of residual insertion distance to a previously identified lesion, informing scope advancement without repeated fluoroscopic confirmation. Second, because δ distinguishes capsule-only displacement from bulk patient or array motion, it can flag when an apparent position change reflects true capsule movement rather than patient repositioning—relevant when choosing between antegrade and retrograde approach, since route selection depends on whether the lesion lies closer to the proximal or distal extent of confirmed capsule transit.

The coordinates reported here are array-relative, whereas clinical localization conventionally references the ligament of Treitz as the duodenojejunal landmark separating proximal from distal small bowel. Reconciling the two requires a one-time registration step: if the array’s position relative to the ligament of Treitz is established at the start of the procedure (e.g., via a brief fluoroscopic or palpation-based landmark check), subsequent array-relative position estimates can be transformed into a ligament-of-Treitz-referenced coordinate via a fixed offset. This registration step is not evaluated in the present study and is identified as necessary work prior to clinical deployment.

The benchtop array validated here is intended to inform a wearable form factor for eventual clinical use: an elasticated abdominal belt or vest embedding the two sensor layers at a fixed 30 cm separation against the anterior abdominal wall, following the mounting concept used in prior wearable magnetic tracking systems [6,19]. A belt or vest, rather than an adhesive patch, is preferred because the two-layer geometry requires a fixed inter-layer spacing that a single adhesive patch cannot maintain across the abdominal curvature. This wearable configuration was not constructed or tested in the present study; translating the benchtop results to a wearable form factor will require characterizing sensor-to-abdomen slip and respiratory-motion coupling, both noted in Section 5 as sources of geometry uncertainty beyond the tested displacement range.

The experiments used a laboratory-built surrogate—an N52 neodymium magnet (10×12 mm cylinder; 5×5 mm for hardware generalization trials)—rather than a commercial capsule endoscope, consistent with prior work from our group [9]. This choice isolates the magnetic localization problem from capsule-specific electronics but leaves open how results transfer to a specific commercial device. Magnetic moment scales with magnet volume and remanence; so, a smaller or weaker capsule magnet would proportionally reduce field strength and the effective sensing range at fixed SNR gating thresholds (Equation (3)), while a larger or higher-remanence magnet would extend it; the layer-conditioned estimation and SNR-weighted fusion architecture itself is agnostic to magnet strength; only the sensing-volume boundaries and SNR gate calibration would require adjustment for a different capsule’s field strength.

### 5.3. Limitations

Six primary limitations apply, and each defines a concrete next step rather than a closed gap. The robotic arm was selected specifically because it provides repeatable, sub-0.1 mm ground-truth trajectories needed to quantify positional and orientational accuracy against a known reference—a guarantee that a phantom or in-body trial cannot offer without introducing its own tracking uncertainty. Continuous random motion and rotational tumbling, discussed further in relation to NN generalization in item 4 below, are consequently not represented in the present validation. First, benchtop validation uses rigid-body step displacements (1.00–4.00 cm) that instantiate asymmetric visibility but do not replicate circumferential occlusion mechanics or physiologically shaped peristaltic waveforms; phantom validation against a synthetic bowel model with controllable wall motion is the immediate next step, and would additionally allow the framework to be tested against continuous rather than step-function displacement profiles. Biological tissue is weakly diamagnetic ($\mu_{r}\approx 1$); so, field distortion from tissue itself is expected to be negligible; the dominant unmodeled error sources are physiological motion (respiration, peristalsis) and wearable-array slip relative to the abdomen, both of which introduce geometry uncertainty beyond the tested 1.00–4.00 cm displacement range, along with clinical EMI sources (e.g., powered beds, nearby imaging equipment) not present in the benchtop setup. Second, static ellipsoid calibration cannot compensate dynamic EMI from ferromagnetic infrastructure; adaptive online calibration that re-estimates $b^{\left(l\right)}$ and $M^{\left(l\right)}$ on a sliding window of recent measurements [24] is immediate future work, and the per-sensor SNR gate developed here would directly support such an extension by identifying which measurements are trustworthy enough to include in the re-estimation window. Relatedly, the reported accuracy figures are instrument- and environment-conditional: Earth’s static magnetic field is absorbed into the per-layer hard-iron bias term $b^{\left(l\right)}$ during ellipsoid calibration (Section 3.5) rather than characterized independently, and a magnetometer with different noise floor or resolution than the LIS3MDL used here would shift the absolute error values reported. Third, the 6.15% non-convergence rate at the lateral boundary may require array enlargement for wide abdominal profiles; the coverage design methodology in the companion study [21] provides a patient-specific sizing pathway, though enlarging the array trades coverage for the inter-layer separation that currently governs the conditioning advantage in Table 3. A systematic sweep of sensor count against Jacobian conditioning was not performed in this study. However, the array geometry itself was derived from a systematic coverage-based sweep over intra-layer (5–20 cm) and inter-layer (25–35 cm) spacings, reported in our companion IEEE Access study [19], which identified 15 cm intra-layer/30 cm inter-layer spacing as coverage-optimal. A dedicated sweep linking array geometry directly to Jacobian conditioning (rather than coverage) is identified as future work. Fourth, the NN initializer was trained on a single robotic trajectory distribution and has not been evaluated on natural capsule transit including orientational tumbling; online learning that updates the network from in-field convergence outcomes is a parallel future direction. Fifth, the δ index was validated under translational rigid-coupling only; rotational array motion and wearable partial decoupling were excluded and the AUC of 0.924±0.045 is an upper bound that should be re-established once wearable strap-mounted data is available. Sixth, validation used a bare N52 magnet surrogate rather than a complete capsule assembly; capsule-housing polymers are non-ferromagnetic and expected to have negligible effect on the dipole field, but internal electronic components (battery, imaging sensor, wireless transmitter) may introduce localized soft-iron or eddy-current effects not captured by the present ellipsoid calibration, which was fit to the bare-magnet field geometry. Quantifying this effect requires field mapping with a fully populated capsule prototype, which we identify as a necessary step prior to in vivo deployment alongside the phantom validation described in item 1.

## 6. Conclusions

A two-layer magnetometer array architecture was presented that explicitly addresses asymmetric sensor visibility as a motion-induced failure mode via three new contributions: per-sensor SNR gating, manifold-constrained 5D Levenberg–Marquardt estimation via explicit spherical orientation parameterization, and geodesic SLERP orientation fusion weighted by per-layer SNR. Combined with layer-wise ellipsoid calibration and neural network initialization, the framework achieves 2.32±0.35 mm positional and 2.15±0.88° orientational error at 12.4 Hz using eight LIS3MDL magnetometers, validated across 150 independently sampled Monte Carlo trials (93.85% convergence, 95% binomial CI [0.912, 0.965]), with an ablation study confirming that all three algorithmic contributions provide individually significant accuracy gains. The framework maintains < 3 mm error under 1.00–4.00 cm displacement events—a greater than 70% error reduction over single-layer and layer-agnostic baselines—and generalizes across three magnetometer architectures spanning an order-of-magnitude difference in sensor resolution and noise floor. Reported accuracy is specific to the instrumentation and benchtop conditions used in this study; phantom validation under realistic EMI conditions is the immediate next step, with adaptive online calibration and NN online learning as parallel future directions.

## Figures and Tables

**Table 1:** Stratified neural network initialization accuracy on the withheld test set ($N_{\mathrm{test}}=3600$). Raw: NN output prior to LM refinement. Final: after manifold-constrained LM refinement (complete framework). High polar inclination is an orientation-based subset overlapping both spatial zones, not a disjoint partition.

Stratum	n	Raw Pos. (mm)	Raw Ori. (°)	Final Pos. (mm)	Final Ori. (°)
Overall	3600	4.18±1.05	3.82±1.14	2.32±0.35	2.15±0.88
Central Zone	2160	3.24±0.72	2.95±0.81	2.08±0.28	1.92±0.65
Displacement/Boundary	1440	5.59±1.21	5.12±1.35	2.68±0.41	2.49±0.98
High Polar Inclination	520	4.85±1.10	4.64±1.22	2.45±0.38	2.28±0.91

**Table 2:** Per-frame computational timing on AMD Ryzen 7000 under MATLAB R2023b. Instrumented component sum is 44.1 ms; OS/MATLAB scheduler and serial buffer overhead accounts for the remaining ∼36.5 ms, yielding an 80.6 ms wall-clock frame period and 12.4 Hz measured end-to-end throughput.

Component	Time (ms)
I^2^C data acquisition	22.5
NN-based initialization (5D)	1.8
Per-sensor SNR gating	0.1
Manifold-constrained LM (per layer)	18.4
Calibration & projection	1.3
**Instrumented component sum**	**44.1**
OS/MATLAB scheduler & serial buffer overhead	∼36.5
**Wall-clock frame period**	**80.6**
**Measured throughput**	**12.4 Hz**

**Table 5:** Hardware generalization results (N=30 trials per configuration, identical trajectories across all three sensor types). Superscript ∗ denotes statistically significant difference from the proposed method (Wilcoxon signed-rank, Bonferroni-corrected, α=0.05).

Configuration	Method	Pos. Error ± STD (mm)	Ori. Error ± STD (°)
LIS3MDL (N52, 5 × 5 mm)	One-Layer	8.15±1.15 ^∗^	7.55±1.45 ^∗^
	Layer-Agnostic	7.25±1.05 ^∗^	6.85±1.35 ^∗^
	**Proposed**	2.43±0.38	2.11±0.78
LIS2MDL (N52, 5 × 5 mm)	One-Layer	8.45±1.25 ^∗^	8.05±1.55 ^∗^
	Layer-Agnostic	7.55±1.15 ^∗^	7.05±1.45 ^∗^
	**Proposed**	2.55±0.42	2.25±0.95
MMC5603 (N52, 5 × 5 mm)	One-Layer	8.05±1.10 ^∗^	7.45±1.35 ^∗^
	Layer-Agnostic	7.25±1.05 ^∗^	6.75±1.25 ^∗^
	**Proposed**	2.42±0.38	2.18±0.92

**Table 6:** Expanded ablation study results (N=30 independent test trajectories), isolating the three new contributions of this paper (C4: per-sensor gating; C5: manifold-constrained LM; C6: geodesic fusion) alongside previously established components. Superscript ^∗^ denotes statistically significant improvement over the preceding configuration (Wilcoxon signed-rank, Bonferroni-corrected, α=0.05).

Cfg	Key Change	Fusion	Pos. (mm)	Ori. (°)
C1	Full-array LM, global calib., rand. init.	Linear avg.	8.55±1.72	7.22±2.45
C2	+Layer-cond. decomp.	Linear avg.	6.02±1.55 ^∗^	4.55±1.38 ^∗^
C3	+SNR-weighted fusion	SNR-weighted	3.85±1.25 ^∗^	3.05±1.15 ^∗^
C4	+Per-sensor SNR gating	SNR-weighted	3.41±1.08 ^∗^	2.78±0.95 ^∗^
C5	+Manifold-constrained LM (5D)	SNR-weighted	3.02±0.92 ^∗^	2.48±0.82 ^∗^
C6	+Geodesic SLERP fusion	SLERP	2.85±0.75 ^∗^	2.31±0.71 ^∗^
C7	+Ellipsoid calib. + NN init.	SLERP	2.32±0.35 ^∗^	2.15±0.88 ^∗^

**Table 7:** Initialization strategy comparison on the canonical N=30 withheld test set, with all other framework components fixed at C6 (per-sensor gating, manifold-constrained LM, geodesic SLERP fusion, layer-wise ellipsoid calibration). Wilcoxon signed-rank test, Bonferroni-corrected vs. MLP: ^∗^ *p* < 0.005.

Initialization	Pos. Err ± STD (mm)	Ori. Err ± STD (°)	Conv. Rate
Random	2.85±0.75 ^∗^	2.31±0.71 ^∗^	88.5%
*k*-NN (k=1)	2.75±1.15 ^∗^	2.85±1.45 ^∗^	89.1%
*k*-NN (k=3)	2.55±0.95 ^∗^	2.65±1.25 ^∗^	92.4%
**MLP**	2.32±0.35	2.15±0.88	**98.2%**

**Table 8:** Comparison with prior magnetic localization systems for capsule endoscopy. Dynamic: validated under relative capsule–array motion. Asym. Vis.: asymmetric visibility explicitly addressed. κ regime: Jacobian condition number under asymmetric displacement. ?: performance under dynamic conditions unknown (static-only validation). ^†^ Wearable-compatible architecture; primary validation conducted on benchtop platform. ^‡^ Dynamic validation under magnetic actuation, not GI-motion-style relative displacement or asymmetric-visibility testing; position error is the mean of reported axis-wise RMSE (x/y/z).

Work	Sensors	Err. (mm)	Dynamic	Asym. Vis.	Wearable	κ Regime
**Proposed**	**8**	2.32	✓	✓	✓ ^†^	∼20–47
Vedaei & Wahid [9]	9	3.5	✓	×	×	N/A
Song et al. [7]	8	1.4	×	×	×	?
Zeising et al. [8]	16	∼3	✓	×	✓	N/A
Liu et al. [12]	96	∼2.4	×	×	×	?
Shao et al. [6]	16	10.0	×	×	✓	N/A
Han et al. [23]	40	1.1	✓ ^‡^	×	×	N/A

## Data Availability

The experimental datasets generated and analyzed during this study are available from the corresponding author upon reasonable request.
